# Exploring spirituality, religion and life philosophy among parents of children receiving palliative care: a qualitative study

**DOI:** 10.1186/s12904-024-01345-2

**Published:** 2024-02-15

**Authors:** Pau Miquel, Ignasi Clemente, Mario Ciccorossi

**Affiliations:** 1 Spiritual and Religious Care Service (SAER), Palliative Care and Complex Chronic Patient Service (C2P2), Hospital Sant Joan de Déu, Esplugues de Llobregat, Barcelona, Spain; 2 Department of Anthropology, Hunter College, City University of New York (CUNY), New York, NY USA

**Keywords:** End of life, Terminal illness, Spiritual assessment, Relational, Transcendent, Interview data, Interpretative phenomenological analysis

## Abstract

**Background:**

Few studies have examined the spiritual environment of parents of children receiving palliative care in Southern European countries, which are mostly characterized by secularization (or the abandonment of traditional religiosity) and an increase of cultural and religious diversities resulting in a much broader spectrum of spiritual and religious beliefs. This study aimed to explore the parents’ own spirituality, religiosity, and philosophy of life in coping with the care of their child with palliative needs.

**Methods:**

Qualitative interviews of 14 parents of children included in a palliative care program in a pediatric hospital in Barcelona, Spain. Inclusion criteria were parents of children who have been cared for the palliative care program for a minimum of 3 months and who displayed a willingness to talk about their personal experiences and gave written consent. Interviews were audio-recorded, transcribed by an independent service, and analyzed on a case-by-case basis using Interpretative Phenomenological Analysis.

**Results:**

The three domains identified were life philosophy, relational, and transcendent. Life philosophy included principles that guided parents’ decision-making, and how the onset of their child’s serious illness had promoted a change in their values. Relational was focused on how they perceived themselves (e.g. motherhood), others (e.g. one’s own child exceptionality), and the way they believed others perceived and supported them (e.g. relatives, friends, and healthcare providers). The transcendent domain involved God-related concepts, divinity and divine intervention (e.g. a miracle as an interpretive framework for that which cannot be explained within scientific knowledge limitations).

**Conclusions:**

Inflexible categories identifying parents as having a particular religious faith tradition are not sufficient to capture the interrelation of knowledges (ethical, religious, scientific) that each parent generates when faced with their child receiving palliative care. Clinicians should explore parents’ spirituality in an individualized way that responds to the uniqueness of their experiential process.

**Supplementary Information:**

The online version contains supplementary material available at 10.1186/s12904-024-01345-2.

## Background

Spiritual needs at the end of life still remain as the most unknown aspect of palliative care. Spirituality and spiritual dimensions of care have recently gained increasing attention, but their potential contribution to palliative care has been largely unexplored, particularly in pediatric palliative care since the majority of studies have been focused on adults [1–3]. On the other hand, data on the relevance of spiritual assessment at the end of life have been mainly reported in studies carried out in Anglo-Saxon countries [4–7]. Given the multifaceted nature of spirituality and the different ways in which spirituality is understood and integrated into end-of-life care across cultures, extreme caution should be taken when transferring approaches from other countries with substantial cultural disparities [2].

In the European setting, indicators of religiosity have generally characterized this continent as the most secularized region in the world [8], with progressive abandonment of traditional religiosity and strengthening pluralism leading to increased cultural and religious diversities. Although Spain has been traditionally a Roman Catholic country, there is currently a high degree of religious diversity and new spiritualities resulting from global immigration inflows over the last two decades [8, 9]. However, despite the presence of these new forms of spirituality and the dramatic fall of practicing Catholics, the Roman Catholic Church continues to have a predominant symbolic, cultural, and political influence on the Spanish public sphere [7]. Within this paradoxical religious context of secularization with new spiritualities and continued public and political influence of the Roman Catholic Church, spiritual services in health care in Spain present some important differences with respect to Northern European countries and the Anglo-Saxon world [1]. In contrast to Northern European countries such as the Netherlands [10], a formal process of standardization and professionalization of hospital chaplaincy tasks and duties has been lacking, mostly due to the agreement between the Vatican state and Spain that attributed to the Roman Catholic Church the predominant responsibility of hospital pastoral care [8, 11]. However, Spain’s progressive secularization process and the increase of cultural, spiritual, and religious diversity demanded new professionals who could provide responses to more complex and individualized spiritual and religious needs [11]. To support such needs, some hospitals have developed new positions of spiritual counsellors. At the hospital where the present study was conducted, spiritual counsellors are part of the hospital’s Spiritual and Religious Care Service (*Servicio de Atención Espiritual y Religiosa*, SAER). The reference framework models of SAER are chaplaincy services in US and UK hospitals [2, 5] as well as the consensus model proposed by Puchalski [3]. Similar to multidisciplinary teams present in other countries (USA and northwestern Europe), spiritual counselling professionals combine different activities including patient care, teaching, training professionals in spirituality, and research. Also, SAER operates within a trans-denominational perspective and is respectful of the various religious confessions and philosophical positions of people to which its services are offered.

In the framework of pediatric care programs, palliative care specialists are aware that spirituality should be an integral part of the services offered to seriously ill children and their families, but little is known about the modes of delivering spiritual care [5]. It has been extensively recognized that parents need support to cope with the life-threatening diseases of their children [12–14]. However, how spirituality and religious beliefs are involved in the integral approach of palliative care remains to be fully elucidated. Improving our understanding of how parents of children with life-threatening conditions draw upon their religious and spiritual beliefs and practices when faced with their children’s diseases is a primary goal for pediatric palliative care clinicians and researchers [6].

Despite attempts made to clarify the implications of spirituality in the field of terminal illness and palliative care, a central challenge for evaluating spirituality in the context of health is how to best define and operationalize religion and spirituality [1, 2, 15, 16]. Hexem et al. [6] proposed an integrative construct of religiosity, spirituality, and life philosophy (RSLP). The integrative construct of RSLP includes the European Association of Palliative Care’s (EAPC) definition of spirituality as “…the dynamic dimension of human life that relates to the way persons (individual and community) experience, express and/or seek meaning, purpose and transcendence, and the way they connect to the moment, to self, to others, to nature, to the significant and/or the sacred” [2]; as well as the concept of life philosophy, which makes possible the theoretical inclusion of parents that vindicate non-spiritual and non-religious ways of living their child’s terminal illness [4]. For this reason, in this study we use the more integrative and broader concept of RSLP, instead of the terms religiosity and spirituality (R/S) that have been commonly adopted in the field of studies of spirituality in health [1]. RSLP provides a more adjusted conceptual framework to examine a context as secularized as is the case in Spain and also has been used to study the experience of parents of serious ill children [6]. The objective of this study was to explore parents’ own religiosity, spirituality and philosophy of life in coping with the care of their child with palliative needs.

## Methods

### Design

The study adopted a prospective qualitative design with Interpretive Phenomenological Analysis (IPA) methodology [17]. IPA is an established methodology in clinical, health and social psychology, which provides a stance and protocol for the analysis of experiential qualitative data.

### Setting

Participants were recruited from the Pediatric Palliative Care Unit of the Hospital Sant Joan de Déu (SJD) in Barcelona, Spain. The hospital is a highly specialized pediatric reference center providing care to local, national, and international patients and families. The SJD Pediatric Cancer Center is the largest monographic pediatric oncological center in Europe. The Pediatric Palliative Care Service has an interdisciplinary team made up of medical doctors, pediatric nurses, psychologists, social workers, and spiritual care specialists.

### Ethical considerations

The study protocol was approved by the Clinical Research Ethics Committee (CEIC) of the Fundació de Recerca Sant Joan de Déu, (code PIC-164-19, approval date 19 September 2019) Barcelona, Spain. The participants were fully informed of the purposes and characteristics of the study, and all participants provided written informed consent. Participants’ responses were kept confidential. Participants were assigned a random numerical code and their identity was revealed exclusively to the authors. Participation in this study was voluntary and refusal to participate did not cause any disadvantages for non-participants.

### Research team and reflexivity

Data collection was carried out by the first author (PM) who is a psychologist with a degree in spirituality and religion, and has worked as spiritual care specialist at the Hospital Sant Joan de Déu for 6 years. IC is an anthropologist specialized in pediatric communication who was involved in data analyses. MC is a theologian and pedagogue specialized in transcultural spirituality who has worked as spiritual care specialist at the Hospital Sant Joan de Déu for 5 years. Although from the same hospital, there was no relationship between study participants and the interviewer prior to the study, except for three cases in which PM, the principal investigator and first author who conducted the interviews, had assisted the parents in his task as spiritual care specialist.

### Sampling strategy

Parents were purposively sampled to ensure representation of a diversity of religions and cultural backgrounds in order to illustrate the diversity of patients and families at the hospital. The sample size corresponded to studies using a similar design [18–20]. Inclusion criteria were parents of children who have been cared for the palliative care program for a minimum of 3 months and who displayed a willingness to talk about their personal experiences as assessed by the palliative care program professionals in charge of their routine care. Exclusion criteria were parents whose children had already died or whose children were in a very critical phase of the disease, as well as parents in a situation of great emotional instability for whom participating in the study could have had negative consequences according to the assessment made by the psychologist of the palliative care program.

### Recruitment strategy

Purposive sampling recruitment took place over the entire period of the research study until data saturation was reached. Religious affiliation is not information that is collected from families to be included in patients’ medical records. Instead, information about a family’s cultural background is collected as a standard hospital practice at the first encounter and at admission to the hospital. Some inferences of a certain traditional religiosity linked to particular cultural contexts can be made nonetheless. However, except for the cases in which the principal investigator (PM) had already met the informant, the PI did not have explicit confirmation of the religious affiliation declared by the person/family until the PI met with the families to initiate the informed consent process. Thirty-three eligible families were identified by their palliative care program team according to the eligibility criteria. The team members requested and obtained permission from the parents to be contacted by the principal investigator (PM). The number of eligible families who were invited to participate was further reduced to 12, due to logistical issues such as losing contact with the families or families no longer receiving care at the hospital (6 families); changes in patient health or death (10 families); and changes in family stability (5 families). Only one family declined to participate. Each of the 11 parents to be interviewed gave consent before the interview was conducted.

### Interview guide

An interview guide for the study was created based on the reference assessment tool developed by the Spirituality Group of the Spanish Palliative Care Society [21, 22], together with widely used questionnaires such as HOPE [23] and FACT [24], and additional questions obtained from published research [25]. The final interview guide (Supplementary Information) included 20 items divided into 8 sections (initial experience at diagnosis; changes in life perceptions; thoughts about the meaning of life; role of religiosity, spirituality and life philosophy; influence of God in their lives; hope-related beliefs; guilty feelings; and advice to other parents in a similar situation).

### Interviews

Between May 2020 and July 2021, recruited parents were invited to participate in a qualitative study based on interviews. All interviews were carried out by the first author (PM). Participants decided themselves whether both parents (father and mother) or the father/mother alone would attend the interview. The interviews were carried out in Catalan, Spanish or English as a foreign language. Face-to-face interviews were audio-recorded using Zoom-H1 handy recorder and the Zoom platform was used for online interviews during COVID-19 restrictions. The transcripts were not returned to participants for comments and/or corrections.

### Data management, coding, and analysis

Interviews were transcribed by a professional commercial service and analyzed on a case-by-case basis. Data analysis adhered to the “six stages” of IPA: reading and rereading of the interview transcripts; initial noting; developing emergent themes; identifying connections across themes; moving to the next case; and looking for patterns across cases [17].

Initial codes developed from the first cases informed the analysis of subsequent cases. In keeping with the iterative process of IPA, as new codes were generated in later transcripts, earlier transcripts were reviewed and tentatively modified. Latter stages of the analysis focused on identifying connections between codes and patterns across cases. Clusters of codes were then organized into three superordinate areas defined as domains.

Preliminary coding was conducted by the first author (PM). Emergent codes were discussed by the other authors during regular meetings. All members checked that the interpretations were appropriately grounded in the data. The qualitative data analysis software ATLAS.ti 2.0 was used to organize data while also retaining the richness and complexity of each participant’s contribution. In the final written report, efforts were made to include quotes from a variety of participants while also illustrating the nuances of particular cases within each theme.

### Rigor

We created a credible interpretation of the data by continuing data collection until data saturation, and by having the three authors independently analyze the data until reaching consensus [19]. An additional measure of quality for IPA research was providing adequate evidence for each theme according to the size of the sample. The authors acknowledge that their own beliefs and interests in applications of phenomenological ideas to health psychology, as well as their experiences in palliative care could have influenced the interpretation of the data. While a relativist position was used with regard to data collected, the analysis was framed by both a relativist and realist position [20]. Yardley’s characteristics of sensitivity to context, commitment and rigor, transparency and coherence, and impact and importance were carefully considered as markers of quality throughout the analysis [26].

## Results

### Clinical and demographic characteristics

A total of 14 parents of 11 children receiving palliative care took part in this study. This sample size was within the upper limit of the number of participants advocated for studies employing IPA [17, 27]. Demographic and clinical data of participants and their children are shown in Table 1. The mean age of the parents was 36 years (range 24–47 years) and the mean age of their children was 5 years (range 1–12 years). Three children died after the interview.

**Table 1:** Demographics and characteristics of parents and children

Characteristics	Number of cases
Parents	14
Age, years, mean (range)	36 (24–47)
Gender	
Male	3
Female	11
Relationship with the child	
Father	3
Mother	11
Religion	
Roman Catholic	5
Muslim	3
Orthodox Christian	2
Church of England	1
Non-religious	3
Parent’s birth place	
Spain	4
Eastern Europe	4
Other European countries	2
South America	3
North Africa	3
Diagnosis of the child	
Neurological disease	6
Perinatal	3
Cancer	1
Heart disease	1
Age of the child, mean (range)	5 (1–12)
Age of child at diagnosis	
Prenatal	2
At birth	5
Before 1 year	2
Between 5 and 10 years	2

### Code identification and domains

The codes identified allowed us to define the following three domains: life philosophy, relational, and transcendent. The classification into these three main areas was intended to provide an explicative framework to sensitize healthcare professionals in the detection and assessment of the religious and spiritual contexts of parents of children receiving palliative care.

### Life philosophy domain

In the life philosophy domain (10 codes), we have included moral and vital principles, guides or precepts used by the informants to guide their decision-making around their child’s care and the interpretive frameworks with which they sought to create meaning in their lives (Table 2). A first theme in many interviews involved a theory of origin (code 1.1) (e.g. why?) as parents grappled to find a reason or a cause that explained the origin of their child’s illness. In this process of searching for the origin, parents explored the different sources of knowledge (code 1.2) (scientific, clinical, religious or differentiated knowledge that parents have developed of their own child) as they looked for hope, potential treatments, or some type of answer. Parents referred to the fact that the current available knowledge was insufficient to cure their child and, therefore, described themselves as being in a liminal state with respect to all these knowledges.

**Table 2:** Characteristics of the three domains of life philosophy, relational and transcendent

Domain	Codes	Description
1. Life philosophy	1.1. Theory of origins	Parents’ explanations of the reason of what caused their child’s illness
	1.2. Sources of knowledge	Research process for sources of knowledge that can explain their child’s illness
	1.3. Liminality	Feeling between the borders of knowledges, at the limits of the various available knowledges
	1.4. Uncertainty	Not knowing what their child’s future will be
	1.5. Ethical limits	Setting of ethical limits that guide their decision-making in regards to their child
	1.6. Change in values	Reorganization of personal and family values as a result of caring for their child
	1.7. Learnings	Incorporation of new ideas and principles into their philosophy of life because of their lived experiences
	1.8. Meaning	Meaning-making process since the irruption of their child's illness
	1.9. Normalcy	Awareness of being part of a minority, which escapes the criteria of normality
	1.10. Images of death	Ideas and representations about what happens when someone dies (postmortem, afterlife)
2. Relational	2.1. Child description	Description of the personal and physical characteristics of one’s child
	2.2. Social support	Help of friends, family, and/or its absence
	2.3. Purpose of child’s life	Possible purpose of the life of their child
	2.4. Child as a source of knowledge	How their child's reactions help parents know how to act, think, or make a decision
	2.5. Others’ gaze	Thoughts and emotions generated by the gaze of others towards their child
	2.6. Relationships with healthcare professionals	Relationships (good/bad) between professionals and parents
	2.7. Parental role	Uniqueness of their motherhood and fatherhood
3. Transcendent	3.1. Role of faith	Effect that religious faith has had on their experience
	3.2. Images of God	Conceptualization of divinity and vision of divine intervention in their particular life (and their absence)
	3.3. Prayer	Orientation, content, characteristics and benefits of prayer
	3.4. Hope	Confidence in the future
	3.5. Own spirituality	Personal synthesis on transcendent aspects
	3.6. Miracle	Explanation of a phenomenon from the viewpoint of a transcendent interpretative framework as a way of explaining that which cannot be explained from a rational viewpoint
	3.7. Rituals	Religious or non-religious practices they have performed with/for their child
	3.8. Religious metaphor	Expressions from sacred books used as an analogy of their personal situation and experiences

*They told me that when he would be born he would die, and now he's two years old and he's still with me, although he has many problems, but he's still with me. – LIMITS OF KNOWLEDGE (code 1.3)*

*... because they told me that my daughter would live six months, afterwards [they told me that] she would live a year and today [some doctors] cannot believe that she is [still] alive. – LIMITS OF KNOWLEDGE (code 1.3)*

*... imagine that three years ago they were saying at the hospital that he was going to die that week and it turns out that four years have already gone by [since] (...) you never know. – LIMITS OF KNOWLEDGE (code 1.3)*

A second theme involved parents’ confrontation with the limits of all the available sources of knowledge, which promoted living with uncertainty (code 1.4). However, uncertainty related to prognosis was experienced in two different ways. On the one hand, some parents limited their focus on the child’s needs at the current moment; but on the other hand, some parents gave priority to what it was known, focusing on the fact that they would accompany their child at all times no matter what (code 1.5).

*We don't know where Peter will take us, nor what will happen, nothing at all. – UNCERTAINTY (code 1.4)*

*What we do know is that we will be by his side, that we will take care of him. – UNCERTAINTY (code 1.4))*

*For me [at the beginning] it was waiting and nothing, not knowing anything, now after so much time... I would have liked a little more clarity [information]. - UNCERTAINTY (code 1.4)*

*Then your whole life is uncertain (...) and you deal with it, like [it becomes] a normal thing. – UNCERTAINTY (code 1.4)*

A third theme was the claim of having a distinct and specific knowledge of their child as they were his/her mother. They explained having a maternal knowledge (code 1.8.) based on intuitions that were later on validated by healthcare professionals. Mothers reported having experienced signs prior to any diagnosis or signs that told them something about the development of their child’s illness. Some mothers expressed that at certain times they had more confidence in their knowledge as a mother than in what the doctor could say based on clinical knowledge (code 1.2.).

*The diagnosis came four and a half years after knowing that my daughter was sick. It was a tremendous shock, but it was like actually saying: I was right. – SOURCES OF KNOWLEDGE (code 1.2)*

*Because I always had more faith in my son, in him, in what I saw in front of me, in the person I knew, than in the knowledge of a doctor. – SOURCES OF KNOWLEDGE (code 1.2)*

Another theme was a change in values (code 1.6.) resulting from the experience of caring for a child receiving palliative care. Detailed descriptions were collected in reference to having learned multiple life lessons which had caused axiological changes and had dramatically modified their worldview (code 1.7.). In some cases, parents expressed a deep break with their previous way of life and an in-depth reorganization of their personal value system. There were different factors affecting a change of values, such as the acceptance of the reality of the prognosis, the role played by love in how they cope with their child’s illness, or the peace of mind of knowing that they were in good hands (i.e., their child was receiving the best care in the best medical center).

*My philosophy of life maybe lies in having seen [so much pain]. In other words, the change of vision, after so much pain. – CHANGES IN VALUES (code 1.6)*

*The problems that we thought were problems, one day they stopped to be. I focused all my energy on what really matters, that she is my daughter (...) that has completely changed me. – CHANGES IN VALUES (code 1.6)*

*It’s changed very much because all the time is for Mileva, everything I’m doing is for Mileva. Mileva changed my life and my vision about believing in God. –CHANGES IN VALUES (code 1.6)*

Furthermore, several parents reported feelings related to the confidence that the future may bring good things. They expressed trust in a rebalancing of the world, after bad luck comes a good future. They also trusted that what they had learned would allow them to continue carrying out a process to find meaning in their child’s life (code 1.8.). Parents distinguished three experiences in the meaning-making process: the meaning of their child’s life, the meaning that their child’s illness had in their lives, and the question of the meaning of life in a more global perspective.

*What is meaningful is to accompany her to her destination, which is what she was created for [her daughter to return to God]. –MEANING (code 1.8)*

*The meaning of life for me is to be able to keep going and to be able to endure the burden.–MEANING (code 1.8)*

*The other thing is the confidence I have in the world rebalancing itself (...) that is, that everything happens for a reason. – MEANING (code 1.8)*

### Relational domain

This domain (7 codes) includes aspects related to identity, parental role, ethical and moral social models around how the informants relate to themselves, to others, and to the view that others have or project on them (Table 2).

A first code involves the conceptualization they make of their child, including their child’s personality, abilities, and limitations with respect to normal childhood, from which they feel that they are outside (code 2.1). Some mothers also described the central place their child occupied in the family system, being the recipient of the concerns and attention of their immediate family circle and friendships (code 2.2). Other mothers also referred to the relationship they established between the meaning of their child’s name and the child’s characteristics and personality, making explicit statements about the purpose or meaning of their child’s life (code 2.3).

*The one who is tied to a wheelchair is my daughter, the one who is losing her childhood, the one who is losing everything is my daughter, I have everything. – CHILD DESCRIPTION (code 2.1)*

*Actually, she is now trying to... not to walk, but she’s trying to put her feet on the ground. And she is trying to say some, some words, just not words, just like sounds. I like these things but I really know that Mileva [will] never be, we’ll never be the normal baby like other babies. – CHILD DESCRIPTION (code 2.1)*

Another code that was found in several mothers’ interviews was the vindication of the possibility of access to the happiness of their child despite their illness, how others saw their child, and what others projected on their child. For some informants, the experience of happiness was linked to the possibility of seeing their child’s smiling. In four informants, their child’s smiling was described as the source that allowed them to forget their own suffering and contributed to giving meaning to their experience of parenthood.

*He gives me strength, him, him. When I see him smiling and happy and content, I forget everything. – CHILD AS A SOURCE OF KNOWLEDGE (code 2.4)*

*He is the happiest child I know on the face of the earth. –CHILD AS A SOURCE OF KNOWLEDGE (code 2.4)*

*I'm eager to see how my daughter is happy with everything she has, I already see that she's happy because she's loved, right? –CHILD AS A SOURCE OF KNOWLEDGE (code 2.4)*

A further aspect was the importance given by a large number of participants to the effect that had on them as parents how other people they encountered on the street literally gazed at their child (code 2.5). Parents expressed that others’ gaze projected their children as only experiencing unhappiness, suffering, and limitations. Some parents reported suffering because of how society came to look at their children, and were critical of current social values that emphasized abilities and success and placed outside of society those who did not fit. Some parents acknowledged that they understood how others’ gaze was less compassionate than their own, since they also used to look at seriously ill children that way, but now that their own child was seriously ill, their gaze of seriously ill children had completely changed. Parents also valued when the professionals’ gaze towards their child was similar to the way they saw their children.

*Others just think he must be suffering, poor thing, but that's not the case, we know he's a happy child. – OTHERS’ GAZE (code 2.5)*

*When he's your son, you look at him for his abilities. – OTHERS’ GAZE (code 2.5)*

*When I'm walking down the street and I see a disabled child in a wheelchair, I can understand the families who have a lot of strength to accept that and support their children (...) it also happens to me when I'm on the bus and he shouts "ah, ah, ah..." and everyone is looking at the boy, but I accept that, because it's true that sometimes people don't really approve, but I don't care because he's my son and I have to accept him as he is, do you understand me? – OTHERS' GAZE (code 2.5)*

*It also helps when the professionals don't just see an illness when they look at her, I mean they also see other things [in a more holistic way] – OTHERS' GAZE (code 2.5)*

The centrality of the social and family support that parents felt they were receiving (code 2.2) was also a relevant argument. Several participants stressed the importance of having a support network of friends and peers who were able to accompany the family in this situation. Such social support was a crucial factor in their coping. Parents recalled the essential support of family and friends that they received from the very start of the illness, and how their understanding, support, and affection have continued to accompany them.

*I'm not religious, I think we've been very lucky, because we've had one hundred percent support (...) and our friends have never stopped inviting us to things and including us in their lives just like before, as always, we've always tried to accept their invitations and to participate, and not use Peter as an excuse for not being able to do things. – SOCIAL SUPPORT (code 2.2)*

*People [friends and family] felt the need to do something more and then I kept asking for things, to light a candle, to send us a drawing or a letter, whatever they wanted, I kept making proposals... and when John was released [from the hospital] we had a big party. – SOCIAL SUPPORT (code 2.2)*

*The whole family loves him, when they call me on the phone the first question is about him, how is he, that's always the case. – SOCIAL SUPPORT (code 2.2)*

Other parents explained that their social network of relationships had been affected negatively because of their child’s condition. In these cases, they experienced loneliness and disappointment with the absence of the reciprocity from friends and family they had expected, which was an ongoing source of difficulty that was challenging and increased their suffering.

*You start to develop a worldview that doesn't fit with many people. – SOCIAL SUPPORT (code 2.2)*

*I have not felt﻿ accompanied in this whole process, not by family or by anybody. They have been selfish and they still are (…) when you go home on leave and you expect to find something to eat dinner, to relax, to feel accompanied, and I go and there is nothing. – SOCIAL SUPPORT (code 2.2)*

*There was no consolation because more than anything we are alone, alone, the only support we had here is the doctor, what is the most difficult thing? Being alone and not being able to do anything about it. – SOCIAL SUPPORT (code 2.2)*

### Transcendent domain

The transcendent domain (8 codes) includes the personal and institutional religious traditions and practices that the informants follow in the context of their child’s care and attention. It also includes a synthesis they have made since the onset of their child’s illness and how it relates to their specific religious tradition (Table 2). The main themes of this domain were grouped into three relevant codes: the role played by faith in parents’ coping (code 3.1.), the image of God constructed by the informants (code 3.2.), and parents’ prayers and religious practices linked to the care of their seriously ill child (code 3.3.).

Regarding the role that faith plays in parents’ experience, some informants referred to their faith as having grown/increased as a result of their child’s diagnosis. This event in their lives had allowed them to see beyond the disease and not be afraid to accept a poor prognosis. Some parents expressed the certainty that for God, suffering had a meaning/purpose that might not be knowable to humans. Other informants, however, expressed a disappointment with their faith (code 3.1.). They hoped to find an answer to their anxieties in religion and reported not finding the comfort they had expected. For two parents, their religious experience as a family was limited to or channeled through the child’s grandparents’ religiosity. Although these parents did not consider themselves to be religious, they had agreed to celebrate rituals such as baptism or communion out of respect for the traditional beliefs of the child’s grandparents.

*Faith for me was a disappointment, I realized that when John was very sick I had no one to turn to. – ROLE OF FAITH (code 3.1)*

*In my case it [faith] is a real experience, not a thought or a set of beliefs or dogmas or norms. For me, faith helps me to live and helps me to be happy and to want more. – ROLE OF FAITH (code 3.1)*

*Having a religion [means] we can think more positively because it is fate, if that child has a disease then it is what it is, you have to accept it. – ROLE OF FAITH (code 3.1)*

*I know that my mother's faith has helped her a lot too, and she in turn has been able to support us better. – ROLE OF FAITH (code 3.1)*

Many parents reported descriptions and characterizations of role that God played in their coping. This is a key theme of the transcendent domain, which included a wide diversity of representations or images of God (code 3.2.). Parents’ images of God were also fundamental to establishing whether these images contributed/promoted spiritual well-being as a protective factor or, on the contrary, they became an additional source of distress. God provided a source of strength for some parents who sought information and searched for hope in new therapies that might make their child’s cure possible (code 3.4). For others, the image of God was of a personal type, with whom the informants established a relationship (and dialogue) that they described in terms of accompanying them through their difficulties. A mother described that she and God had established an agreement of collaboration regarding her daughter’s care. Another mother also expressed the certainty that God would always be by her side.

Some parents described how God intervened in their lives directly, how God became the origin and the end of everything that happened, including the concepts of a predetermined sense of fate as well as illness as a test of endurance. Several parents reported that in the face of the uncertainty they were living, they relied on the power of God who had the last word in terms of their child’s fate. For these parents, God was attributed with the power to give both good and bad things, God placed challenges in one’s path, but at the same time, God provided one with the conditions to be able to solve them.

*Allah is happiness, love, is everything. God is everything to me, he gives us strength, he gives us peace, and he gives us all the good things. Also the bad ones. It's like an exam to see if we're going to pass it, right, like a test. – IMAGES OF GOD (code 3.2)*

*I believe that they are tests that [God] gives me, everything, to see how far we are able to keep fighting. God places tests in our way, everything... to see if we are strong enough to endure everything and we manage to keep going. Because God is also there [with you] and gives you things to face it [the test]. – IMAGES OF GOD (code 3.2)*

*Our religion says that if God has given you something in life, then it is because he will surely give you something when you die, he will reward you, and that helps, of course. – IMAGES OF GOD (code 3.2)*

*God is a DJ of the faith list. – IMAGES OF GOD (code 3.2)*

The use of prayers and religious practices was described by several parents as important tenets of their religiosity in their sick child’s daily care (code 3.1). Some parents placed images of saints from their religion around the child’s body during hospital admissions. One parent recalled repeating the same prayer when her child suffered a convulsive crisis until the crisis ended, and this helped her to stay calm. Another mother described prayer in a similar fashion, as a way of calming down when experiencing anxiety and pain. Faced with despair, she asked God to help her calm down, asking God to give her strength and serenity.

For some mothers, religiosity had been transmitted from grandparents to grandchildren, where grandparents prayed with the sick child. Some parents expressed that they preferred to use their own prayer, made up of their own words, rather than using traditional prayers from their religious tradition, which they felt were too far removed from their internal experiences (code 3.3). These parents underscored the central place that their child had in all their appeals for God’s help. Additionally, a few parents also explained the “power” of knowing that their child was present in the prayers of religious communities, relatives, and friends and that many people around the world were praying for her/him/us (code 3.3).

*…people we didn't know were praying for Laura. It filled us with happiness. We knew that people at a specific time, everywhere were praying for Laura. So this made us feel that there was a concern for Laura and that God was really with her, you know? – PRAYER (code 3.3)*

*…as a family we pray at home, we always pray first for him, second for him, third for him... giving thanks always for what God has given us. – PRAYER (code 3.3)*

*Yes, I’m praying, but I’m not only depending on God. I don’t think that I should put my hands down and wait for God [to do] what he may do (…) I think [that] me and God are collaborating, I’m always speaking with him. – PRAYER (code 3.3)*

The integration of life philosophy, relational, and transcendent domains into the construct of RSLP based on the description of two individual cases is shown in the Supplementary material 2.

## Discussion

Despite reports recommendations issued by Spanish health authorities [28, 29] underscoring the need for spiritual support for patients and families, no clear approach has been put forward nor the needs and concerns of parents of children requiring palliative care have been adequately identified and addressed in the context of this Southern European country. Therefore, the main goal of the study was to explore RSLP of parents of children with palliative needs in Barcelona, Catalonia, Spain. We have examined how parents strived to make sense of what was happening, reorganized their worldviews and values after the onset of their child’s life-threatening/shortening illness, and learned to coexist with an unpredictable future and the prospect of their child’s death.

The present findings make clinically relevant contributions to redefining the role of spirituality and religion in the pediatric palliative care setting. We would like to highlight the spiritual needs of non-religious parents in a predominantly secular sociocultural context, and the importance of relational aspects, such as parents’ perceptions of professionals’ gaze of his/her child and their perception of the support that they receive from families and friends. We would also like to highlight three additional key findings. First, some parents reframe uncertainty as potentially engendering hope in the face of a poor diagnosis. Second categories associated with traditional religious denominations are insufficient to account for the unique and ongoing meaning-making process that each parent of a child with palliative needs engages in (i.e., Who are we now? What is happening to us?). Third, the concept of miracle is identified as an interpretive framework developed by parents in response of that which cannot be explained within the limitations of the evidence based on the currently available scientific knowledge.

The explanatory model of the present study is consistent with three dimensions of spirituality (intrapersonal, interpersonal, and transpersonal) set forward in international consensus definitions [2, 3], as well as in questionnaires and total care models developed mainly in adult palliative care and validated for the Spanish context [22, 30]. Our contributions also make possible to add complementary data to these three dimensions of the consensus definition of spirituality and their three associated experiences (meaning, connection, transcendence). The proposal of more specific and narrower codes within each of the three dimensions based on parents’ unique experiences in terms of RSLP will be useful drivers for the assessment of spirituality in the specific context of pediatric palliative care. It is beyond the scope of this article to examine in detail all the codes listed in Table 2. Nonetheless we would point out that some of these new codes such as theory of origins, experience of liminality, images of death, role of others’ gaze, idea of miracle, and images of God, have the potential of establishing specific goals in the field of spiritual care in a transdisciplinary approach [1–3, 31] providing a complementary framework to previous research [4, 6, 7, 32, 33] especially in order to improve the main models of assessment used in palliative care in Spain [21, 30].

## Main findings

### Life philosophy domain

This domain shows how parents hold different (and often contradictory) sources of knowledge as they seek answers to questions about the origin of their child’s illness, its causes, their child's prognosis, and their and their child's future prospects. These concepts are in line with the intrapersonal dimension of the consensus definition of spirituality [3], although we expand the EAPC definition of the meaning dimension [2, 34] by proposing three new codes based on parents’ experiences: theory of origin, experience of limits of knowledge, and uncertainty.

Parental statements included in the “theory of origin” code illustrate how parents’ RSLP is interrelated with Fisher’s analysis of scientific explanations and with Sitaula’s analysis of genetic counselors’ roles, where religious and scientific explanations coexist in a background of high vulnerability and uncertainty [13, 35]. Our findings also contribute to improve the understanding of how spirituality is related to decision making, to prenatal genetic counseling, to future medical decisions, and directly or indirectly to parents’ trust in clinical teams [6, 13, 35–37].

Parents’ RSLP statements also reveal a dialogue between different sources of knowledge (scientific, ethical, religious, clinical, spiritual, parental) from which answers were sought in three interrelated meaning-making processes: the meaning of the disease, the meaning of the life of their own child, and the meaning of life more globally. This triple dimension of meaning expands and complements the main spirituality evaluation models in the field of palliative care such as HEXCOM and the GES model that have been proposed for Spain [21, 22, 30].

In the absence of medical or scientific knowledge that can provide clear diagnostic and prognostic answers, or more plainly, a degree of certainty, there is indeed great suffering [13]. In our study, we observe also how such a lack of knowledge about prognosis also mobilizes parents to look for answers beyond spiritual coping in the direction of establishing a new normality [14, 38]. Parental knowledge based on the experience accrued over years spending uncountable hours taking care of their own child was qualified by parents as an essential source of knowledge, sometimes more valid than the existing limited medical evidence. In this respect, the uncertainty associated with the limitations of scientific knowledge was not necessarily perceived as a negative circumstance by some parents, as uncertainty kept open the possibility that the child might live longer than what was medically expected. The potential hope of uncertainty may complement perspectives of uncertainty that distinguish between short- and long-term uncertainty that could be a source of worry after diagnosis [7, 13].

### Relational domain

The consideration of the relational domain and social/community benefits as part of the evaluation/intervention within the spiritual aspect of palliative care has been suggested by recent reviews [1, 2, 13, 14, 39]. The relational domain in the present study is similar to the interpersonal dimension of the consensus definitions of spirituality [3] and the experience of connection reported in other models [22, 30]. In our study, the relational domain also includes the parents’ relationship with the child (patient) and healthcare professionals, in addition to the descriptions of significant changes in the parental role, the absence/presence of family and social support as perceived by the interviewed parents, others’ gaze, and the child as a source of meaning or strength.

Despite cultural differences between Northern and Southern European countries reported by research that examines the effectiveness of assessment questionnaires in the interpersonal domain of spirituality [2, 21, 22] our findings do not differ substantially from previous research studies conducted in Anglo-Saxon countries [6, 7, 13]. Therefore, our study is in line with this research underscoring the importance of interpersonal aspects for parents' spiritual experiences of caring. We would also like to highlight the importance of the relationship between parents’ relational domain and their ongoing adaptation to a “new normal,” as parents reassess their relationships and deal with the risks of feeling isolated and lonely, but also with the opportunities to feel supported by family, friends, and their communities [6, 13].

Multiple studies examining parents’ experiences note that parents describe their relationships with clinicians as fragile, which has the potential to have a significant effect on their relational experience [6, 12].Our study indicate that how parents experience clinicians’ gaze (i.e. how clinicians see their children and themselves) also has an effect on their perception and their experience of the humane quality of the compassionate support that they receive from clinicians. Parents are aware that there are differences in how they see their own child versus how others, including clinicians, see him/her. Although parents feel that others see their child as mostly experiencing unhappiness, suffering, and limitations, they also see their child as experiencing happiness and being a source of happiness, strength, and meaning. This difference between how parents see their child and how others see him/her has ethical implications for treatment decision-making [36, 40].

Finally, a key finding of the relational domain is the significance that parents attribute to the process of redefining their parental role of a child with palliative needs. This new identity encompasses both positive and negative changes in family and social support after the irruption of a serious illness. Parents describe this process of creating a new identity for themselves in terms of a form of learning and a change of values as they adapt to this new normality [6, 12, 13].

### Transcendent domain

How parents from a range of religious and secular perspectives cope with the multiple disruptions brought about by their child’s life-threatening condition continues to be an understudied aspect of pediatric palliative care research. Despite such a dearth of research, interpretations of families’ cultural and religious beliefs have the potential to enhance professionals’ understanding of parents’ decisions, goals, priorities, and values [5, 6]. The transcendent dimension defined in our study is similar to the transpersonal domain and the experience of transcendence reported in other models [2, 21, 30, 41]. This domain includes religious and non-religious perspectives and different beliefs, practices, and rituals from transreligious and transcultural perspectives.

The results of this domain were grouped under the codes of role of faith, the concept of miracle, the power of prayer, and the image of God. We found that variations within each code did not match necessarily with parental self-concept of believers (spiritual or religious) or non-believers. The codes image of God and role of faith have been identified as key aspects that support parents’ coping in children with rare diseases [33, 35].

Parents who defined themselves as religious tended to hold a theological philosophy of life, which intersected with their life philosophy. Parents reported both positive and negative changes in their faith after the irruption of a serious illness, which is consistent with previous research [1, 3, 6]. For some parents, their religiosity was a source of strength that influenced their coping experiences [7]. However, traditional religious faith may become a source of conflict in the relationships of a father/mother with him/herself, with God, or between spouses [6, 7]. Thus, faith harbors both a positive or a negative potential vis-à-vis parents’ coping and well-being. Also within the role of faith code, we identified statements from parents who defined themselves as non-religious, who also described transcendent aspects linked to a non-religious spirituality. These aspects, which should be addressed beyond any specific religious tradition [32] are particularly relevant in the culturally diverse and highly secularized context of contemporary Spain.

The concept of miracle was found to be used by parents as an interpretive framework for that which cannot be explained within the limitations of current scientific knowledge. Parents also reported the concept of miracle as a way to find hope when prognosis is poor without negating the prognostic and diagnostic facts [36, 42].

Individual prayer was seen as a tool in the child’s daily care, as a way for parents to stay close to their child in difficult moments or crises, and a means to ask for divine help for their child. Parents reported that prayer facilitated sustaining intergenerational and community connections, as well as made parents feel connected to global community prayer groups and prayer chains that were organized for their child by their own religious communities.

Personal descriptions about the relationship with God were reported by parents as a source of strength, source of companionship, or direct intervention in their lives. Eliciting the characteristics that parents attribute to God is a useful indirect way to carry out a conversational assessment of their spirituality and to identify interpretations that may have negative connotations or increase the burden of the disease [1, 41].

### Clinical perspectives, limitations, and strengths

In light of the fact that spirituality is the least evaluated dimension in the field of palliative care [2], the evaluation and planning of spiritual care should be based on parents’ spiritual concerns and capacities (strengths) [6, 7, 30–32]. Developing conversational models for spiritual in pediatric palliative care has been singled out as a top research priority in a large international survey [43]. Our findings contribute to addressing such a priority by providing an initial step in the creation of narrative assessment tools of spirituality that rely on interview conversational methods. A narrative assessment facilitates the identification of parents’ key RSLP components while capturing the unique and individual context, needs, and concerns [44]. This approach may avoid the limitations of more reductive quantitative methods, and may be a first step to develop a new assessment tool adapted to characteristics of pediatric palliative care in a Southern European Mediterranean sociocultural context. Such an assessment tool would require a much larger number of participants than the number of participants in the present study [45]. Our findings can also be applied to increase healthcare professionals’ awareness and skills in identifying spiritual needs in training interventions [31, 46, 47]. On the other hand, evaluations based on categorical (yes/no) questions frequently prevent parents’ expression of their spiritual needs and concerns [7]. Instead, using spiritual interview/conversational methods makes it possible to include and evaluate in a more open and inclusive manner. This is particularly useful to assess the experiences of nonreligious families, who are not usually offered spiritual support despite manifesting spiritual needs in their experience of coping [7, 31, 32].

A first limitation of our study involves the fact that the study was carried out at a single hospital site, in one of the most secularized areas of Spain, with a small sample size, and with a focus on neurological pediatric patients who tend to have the poorest prognoses, a circumstance that may have had an impact on the experiences described by the interviewed parents. A second limitation is related to the fact that father and mother were interviewed together in three interviews, which may condition the expression of the experiences of the two informants. A third limitation is the problem self-selection bias during participant recruitment, as the participants who voluntarily chose to participate in this study may have been more interested in the topic or more open towards ideas of spirituality and religion. A fourth limitation entails the fact that transcripts were not returned to participants for comments and/or corrections. Instead, transcriptions produced by the commercial/professional transcription service were subsequently reviewed and corrected by PM and IC, who did so independently, reiteratively, and systematically. Furthermore, some excerpts were re-transcribed by the authors with the aim of ensuring the highest degree of transcription reliability**.** A fifth limitation relates to the fact that the study took place during the initial stages of the COVID-19 pandemic. Although these extraordinary circumstances undoubtedly created even more stress for already stressed parents, the lack of a comparison between pre-pandemic and post-pandemic data limits the possibility to assess the impact of COVID-19 on these parents and their ability to care for their seriously ill children, as well as the influence on their RSLPs. Finally, it may be possible that prior relationships of participants with the pediatric palliative care team may have had an influence on the way they responded the questions. However, the specific content of the interview guide was unknown to them, and such potential influence would not necessarily constitute a negative bias in all cases. It is also possible that a prior relationship may have facilitated the openness of the informant to share his/her experience. In this sense, there were no substantial variations in the content, duration or the topics presented in the interviews between those informants who had prior relationships with the interviewer and those who did not.

Despite these limitations, this is the first study that examined the spiritual needs of parents of children receiving palliative care in Spain, identifying new codes that will pave the way for subsequent research, particularly in similar clinical settings. A further strength is the clinical applicability of the explanatory model, as its ease of use could improve the integration of spirituality impacting within everyday clinical practice. Further analysis with the available data from this study could be used in future research and intervention development, such as individual case or cross-case analysis, to explore comprehensively some of the codes identified here.

## Conclusions

The clinical application of the present categories and codes does not require the use of direct questions. Instead, their clinical application can be carried out by listening to parents openly first, and then coding using the theoretical framework proposed here to identify, explore, and assess the spirituality and religiosity of parents of children with palliative needs. Furthermore, the identification of each parent’s key categories and codes may be a first step in setting goals and developing an interprofessional spiritual care plan. The comprehensive/holistic model presented here reaffirms the idea of the trans-disciplinarity of spiritual care, as aspects of social work, psychology, nursing, and medicine are present and intersect within the personal experiences of the interviewed parents. Inflexible categories identifying parents as having a particular religious faith tradition are not sufficient to capture the interrelation of knowledges (ethical, religious, scientific) that each parent generates. Spirituality in the field of pediatric palliative care still continues to be understudied, but understanding parents’ spiritual needs and abilities is crucial to improve spiritual care plans within a comprehensive care paradigm.

### Supplementary Information

**Additional file 1.** Interview Guide.

**Additional file 2.** Selected Case Studies.

## Data Availability

The data analyzed during the current study are available from the corresponding author on reasonable request.

## Abbreviations

RSLP: Religion, Spirituality and Life Philosophy
PPC: Pediatric Palliative Care
R/S: Religion and Spirituality
EAPC: European Association for Palliative Care
SECPAL: Spanish Society of Palliative Care
IPA: Interpretive Phenomenological Analysis
