# Supplementary material for: Exploring spirituality, religion and life philosophy among parents of children receiving palliative care: a qualitative study
Source: BMC Palliat Care. 2024 Feb 15;23:43. doi: 10.1186/s12904-024-01345-2 (PMC10868107; doi:10.1186/s12904-024-01345-2)
Supplement: Supplementary file 1 — Additional file 1. Interview Guide. [file 12904_2024_1345_MOESM1_ESM.docx]

**Supplementary Material 1 -** Interview guide

| **Subject/topic** | **Content** |
| --- | --- |
| 1. Experiences during initial diagnosis | • How did it all begin?  • What do you remember about the moment when you were told the diagnosis (first contact with the Pediatric Palliative Care Unit) (…) Do you remember what was going through your head? What were you feeling? What were you thinking? |
| 2. Changes in life perception | • How do you explain your child's illness/situation to a stranger (e.g. somebody who does not know about your child’s illness)?  • How has your child's special needs transformed your life?  • Has your perception of abilities or limits changed?  • What worries you most about your child's illness?  • What do you think would be the most difficult thing for your child to face? And for you?  • What is important to you? What are your priorities?  • Who gives you strength, hope, patience... during moments of distress? |
| 3. Beliefs/thoughts regarding the meaning of life | • Do you believe there is a purpose in life? Does life have a meaning?  • And your child's? How do you notice it? Can you describe it?  • What does having a child with special needs teach you about life?  • What do you think happens when we die? Do you think there is some form of afterlife?  • Is it a source of anxiety or peace? How do you imagine it? (after life?) |
| 4. Role of religiosity | • Some families have guiding principle, values, which sometimes are spiritual or religious... what are your beliefs, religious, spiritual, or otherwise...?  • Has your religiosity/spirituality or philosophy of life been modified/challenged by your child's diagnosis/illness?  • From your RSLP's perspective, what has been the most difficult thing to deal with? And what has helped you? |
| 5. God’s influence in one’s life | • If you had to give an adjective/name/title to God's role in your life, what would it be?  • Is there someone around you who tells you that they are praying for your child? How does it make you feel?  • Do you make any requests/prayers for your child's illness? Why do you pray? Do you ask for anything? How do you feel when you pray?  • Are you connected to any religious/spiritual community?  • How would you describe your child's spirituality? How do you think s/he lives this experience? |
| 6. Hope-related beliefs | • What good things do you think can happen in the future?  • What serves as a source of guidance, inspiration or protection for you?  • What is your hope? |
| 7. Guilty feelings | • If you could go back in time, what advice would you give yourself?  • Would you change anything? Do you think the current situation could have been avoided? Who? How?  • If an ILE (legal interruption of pregnancy) had been possible during pregnancy, what led you to make the decision that you made? |
| 8. Advice to other parents in a similar situation | • What would you say to someone who has just met a Pediatric Palliative Care team or who have just learned about a serious diagnosis like your child's?  • Is there any motto/aphorism/everyday saying or expression that represents you, or your family, in terms of how you are dealing with the care/situation of your child?  • If you had to give a film or book title to everything you have experienced, are living or will live while caring and raising your child, what would it be? |
| Is there something you would like to say that I haven't asked you? | |
