# Supplementary material for: Exploring spirituality, religion and life philosophy among parents of children receiving palliative care: a qualitative study
Source: BMC Palliat Care. 2024 Feb 15;23:43. doi: 10.1186/s12904-024-01345-2 (PMC10868107; doi:10.1186/s12904-024-01345-2)
Supplement: Supplementary file 2 — Additional file 2. Selected Case Studies. [file 12904_2024_1345_MOESM2_ESM.docx]

**Supplementary Material 2** – Selected Case Studies

By presenting two different explanatory models based on a parent’s specific constellation of codes, we would like to illustrate how each parent generates his/her own interrelation of knowledges (ethical, religious, scientific) and consequently his/her unique Religiosity, Spirituality, and Life Philosophy (RSLP).

**Case 1 – Fatima, Munir, and their son Ahmed**

***Context.*** *Fatima and Munir are the mother and father of Ahmed, a 4-year-old boy suffering from a type of epileptic encephalopathy that causes him to experience significant difficulties in his neurological functioning. For 2 years Ahmed has been receiving care from the pediatric palliative care program at SJD.*

**Philosophy of life domain**

Fatima and Munir describe the origin or beginning of the disease process as the period extending between when they first detected a set of physical warning signs until they received an official diagnosis of the rare syndrome that their son suffered from. They locate the organic causes within a divine plan for Ahmed's life, where the idea of fulfilling a destiny is present. Nonetheless, Ahmed’s father also researches information about his syndrome on the internet, where he explores other sources of knowledge.

Faced with an incorrect prognosis that their child would only live for three days, Ahmed's parents have developed their own interpretation of living with uncertainty. They interpret uncertainty from a perspective of submission to Allah's divine plans, which are unknowable to humans.

The limits of medical knowledge open an avenue for hope. Not-knowing provides space for possibilities, alternative explanations and scenarios, some of which are highly unexpected, such as the fact that Ahmed remains alive with his parents. Faced with not-knowing, Ahmed's parents remark that they do have an ethical knowledge in relation to their son's care. Not-knowing not only makes room for Ahmed to be alive (because the predictions were wrong) but allows the certainty that they will be by his side.

In addition to their ethical knowledge (i.e. they will always accompany their son) and the limited scientific knowledge there exists about their son’s syndrome, Ahmed’s parents describe a religious source of knowledge that makes the incorrect medical prognosis more bearable. Ahmed’s father explains that when faced with a seriously ill child, one may not know why the child has such condition or what may happen, but regardless of that, from his religious perspective, one cannot even consider the possibility of not taking care of a child with such an uncertain prognosis. Ahmed’s parents share their rejection of the idea that euthanasia or abortion would prevent Ahmed from suffering. They consider it outside their moral perspective as practicing Muslims.

They express that a certain degree of uncertainty is more bearable than living without hope. They express that not-knowing also allows them to trust in God. Their hope is not so much placed in Allah's direct intervention, but they place their hope in not-knowing what will happen and in living focused on the day to day. In regards to death, they express the belief of a continuity of life after death, they believe in the idea of final judgment and of a space of paradise as opposed to a space of condemnation, such as hell.

**Relational domain**

Ahmed's parents describe how they are aware that there are other children with a greater affectation than their son, and that despite this, these parents are still content and happy. Fatima and Munir share that they do not hold back and give everything they have to Ahmed as they would any other healthy child. Ahmed's mother explains that her view of families with a functionally diverse child has changed. She understands that others may have a standoffish view of her son because she herself used to view functionally diverse children that way before Ahmed came into their lives.

**Transcendent domain**

Fatima and Munir talk about a heteronomous image of God (a God who gives and takes away) and about notions of reward and re-balancing that tend to occur in life. They describes God as the Merciful one. They hope to be rewarded by God for what they have done in their lives. Fatima describes Allah as a caring figure, who does not keep silent, who watches over their sleep, an omnipotent figure who knows no limit of caring for humans. Fatima recounts that she uses a surah from the Koran that refers to these attributes, whenever her son convulses, and which helps her to remain calm until the convulsion subsides.

**
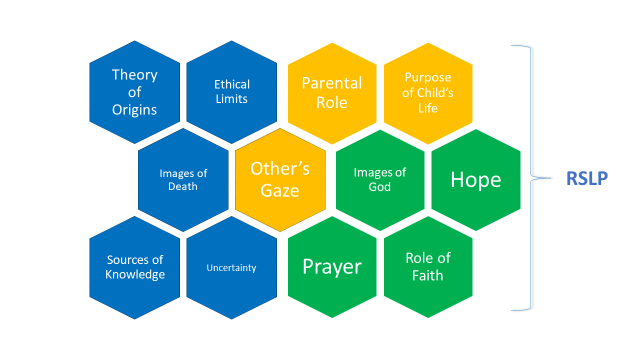
**

**Figure S1.** Explanatory model of Ahmed’s parents’ Religiosity, Spirituality and Life Philosophy (RSLP) according to the life philosophy domain codes (blue), the relational domain codes (yellow) and transcendent domain codes (green) in the experience of caring for their seriously

ill child.

**CASE 2 – Elisabeth and her son John**

***Context:*** *John is a 6-year-old boy suffering from a congenital brain anomaly. Joan was diagnosed a few weeks after he was born. His perinatal period was marked by long hospitalizations and diagnostic evaluations until he was referred to the palliative care service. Elisabeth, John's mother, was interviewed as part of the present study.*

**Philosophy of life domain**

Regarding the origin of the disease, Elisabeth states that neither she nor John’s father had risk factors that would have predicted John’s illness. She believes that everything happens for a reason, emphasizing the importance of one’s stance toward life events and the personal decision one takes consequently. She underscores the value of one’s positive attitude and personal ability to meet life challenges as a way to re-assert oneself, one’s autonomy and one’s personal growth.

She believes that life has a way to balance itself: life gives one what one needs to grow and to learn from the care of a seriously ill child. She remains hopeful that life will balance itself at some point, with good things coming her way after so much hardship.

Elisabeth understands death as a transition. She believes life energy and memories are immortal. She makes a distinction between the material and the immaterial elements of the body. Although she is not sure there exists an afterlife, her preoccupation whether it exists or not has ceased to be a cause of suffering. She expresses that it is easier for her to think about her son's death than her own.

She is understanding of those who decide to terminate the pregnancy of a seriously ill child after the child is diagnosed at a gestational stage. She explains that she would have had an abortion if she had known the suffering that life was about to bring to both her child and herself. She speaks about the injustice or unfairness of the great suffering that a child born with a serious illness will cause both to the child him/herself and his/her parents.

She believes that her son's illness changed her life completely, making her unlearn everything she had learned until then. Her previously life has faded away; friendships, family, identity, life projects and work life have disintegrated. She had to build a new life after such a radical change that turned everything upside down. She states that one learns to live life from a totality/holistic perspective that includes life, death, illness, suffering, and joy. She believes that her axiological change began when she first looked at the world anew with the perspective of someone who had suffered tremendously as a mother.

**Relational domain**

John's mother describes her son as a happy child, while acknowledging that he is outside the realm of what others would consider normalcy. For them, their everyday life with John’s illness has become their normal. She expresses the shock of feeling outside the limits of society’s normalcy, and believes this is the reason why she –the mother of a child who was born chronically ill– has developed a life philosophy that does not fit with the life philosophy of many other people.

In terms of her role as mother, she describes having felt like a “mother-nurse” on many occasions, making decisions beyond her role but which were motivated by wanting to do the best for her child. Regarding how she believes that other people see her, she remarks that her attitude towards John's illness has created a lasting impression on several people close to her, even bringing changes in their lives. She emphasizes the importance of having a supportive network of friends and peers who have accompanied her family throughout their journey.

**Transcendent domain**

Although John’s mother grew up in a Roman Catholic family, her religious faith has not been an important element in how she has coped with her son’s illness.

She feels disappointed by her faith. She had hoped to become more connected to her family religiosity in times of difficulty. Instead, she felt disappointed and lost, having discovered that her religious background did not help with her worries.

She narrates how she rejected a Roman Catholic nun’s interpretation that if her son were to die, his death would be part of God’s plan. Despite her multiple problems with her Roman Catholic background, she still engages in popular religious practices that connect her to her family faith, including devotion to specific saints. She expressed a trust in an afterlife, in the concept that after death there will be someone taking care of her child. Facing her son’s death, she finds comfort in thinking that their ancestors –hers and her son’s –will welcome her child.

**
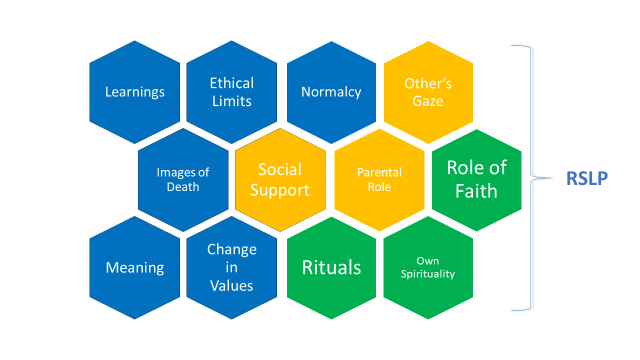
**

**Figure S2.** Explanatory model of Elisabeth’s Religiosity, Spirituality and Life Philosophy (RSLP) according to the life lhilosophy domain codes (blue), relational domain codes (yellow) and transcendent domain codes (green) in the experience of caring for her seriously ill child.
